# IAP antagonists Birinapant and AT-406 efficiently synergise with either TRAIL, BRAF, or BCL-2 inhibitors to sensitise BRAFV600E colorectal tumour cells to apoptosis

**DOI:** 10.1186/s12885-016-2606-5

**Published:** 2016-08-12

**Authors:** Philippos Perimenis, Apostolos Galaris, Alexandra Voulgari, Margarita Prassa, Alexander Pintzas

**Affiliations:** Laboratory of Signal Mediated Gene Expression, Institute of Biology, Medicinal Chemistry and Biotechnology, National Hellenic Research Foundation, Athens, Greece

**Keywords:** IAP antagonists, TRAIL, BRAF inhibitors, BCL2 inhibitors, Synergistic treatments, Overcome resistance in colorectal cancer cells

## Abstract

**Background:**

High expression levels of Inhibitors of Apoptosis Proteins (IAPs) have been correlated with poor cancer prognosis and block the cell death pathway by interfering with caspase activation. SMAC-mimetics are small-molecule inhibitors of IAPs that mimic the endogenous SMAC and promote the induction of cell death by neutralizing IAPs.

**Methods:**

In this study, anti-tumour activity of new SMAC-mimetics Birinapant and AT-406 is evaluated against colorectal adenocarcinoma cells and IAP cross-talk with either oncogenic BRAF or BCL-2, or with the TRAIL are further exploited towards rational combined protocols.

**Results:**

It is shown that pre-treatment of SMAC-mimetics followed by their combined treatment with BRAF inhibitors can decrease cell viability, migration and can very efficiently sensitize colorectal tumour cells to apoptosis. Moreover, co-treatment of TRAIL with SMAC-mimetics can efficiently sensitize resistant tumour cells to apoptosis synergistically, as shown by median effect analysis. Finally, Birinapant and AT-406 can synergise with BCL-2 inhibitor ABT-199 to reduce viability of adenocarcinoma cells with high BCL-2 expression.

**Conclusions:**

Proposed synergistic rational anticancer combined protocols of IAP antagonists Birinapant and AT-406 in 2D and 3D cultures can be later further exploited in vivo, from precision tumour biology to precision medical oncology.

**Electronic supplementary material:**

The online version of this article (doi:10.1186/s12885-016-2606-5) contains supplementary material, which is available to authorized users.

## Background

Colorectal cancer (CRC) is one of the most common malignancies worldwide. Regarding pathophysiology, CRC development is linked to the acquisition of oncogenic mutations. In CRC, like in other tumour types, rational combination treatments can overcome resistance.

Inhibitors of Apoptosis Proteins (IAPs), also known as BIRCs (BIR domain containing proteins) are a class of highly conserved proteins characterized by the presence of Baculovirus IAP Repeat (BIR) domain, a Zn^2+^ ion coordinating protein–protein interaction motif [1], predominantly known for the regulation of caspases and immune signalling. These proteins are crucial for numerous cellular signalling networks. There are eight known mammalian IAPs/BIRCs; among them are cIAP-1, cIAP-2, XIAP and SURVIVIN [1]. IAPs can be regulated by certain endogenous inhibitors of IAPs like the pro-apoptotic protein Second Mitochondria-Derived Activator of Caspases (SMAC/DIABLO) [2–4], a mitochondrial protein released into the cytosol during apoptotic induction [4].

Although several studies have demonstrated elevated levels of multiple IAPs in an array of human cancers, there have been opposing prognostic implications for IAPs in different tumour types, strongly suggesting that the role of IAPs in tumorigenesis is context-and cell type-dependent [5]. Apart from apoptotic-caspase cascade, IAPs are essential modulators of innate immunity signalling, canonical and non-canonical NF-kB pathways as well as TGF-b signalling pathway [6]. Elevated expression of IAPs in certain tumour types has been correlated with tumour survival and resistance to chemotherapy. So, a variety of anti-tumour therapeutics, especially small-molecule inhibitors against IAPs (IAP antagonist compounds (IAC), SMAC-mimetics) are being designed and clinically tested [7, 8]. Since targeting of IAPs can be only partially efficient as anti-cancer therapy, rational combination treatments with other targeted molecules against driver oncogenes, or apoptotic factors can be crucial to overcome tumour resistance.

BRAF is a proto-oncogene which encodes a serine/threonine kinase and regulates the MAP kinase/ERK signaling pathway, which is crucial for cell proliferation. Mutations in this gene have been associated with various cancers, including colorectal cancer (CRC) and display potent transforming activity associated with progression to metastasis [9]. PLX4032 (Vemurafenib) is a BRAF inhibitor which has demonstrated selectivity for the mutated BRAFV600E compared to non-mutated BRAF oncoprotein [10, 11]. PLX4032 was approved by the FDA for the treatment of BRAF-mutated metastatic melanoma [12]. Colorectal cancer and other cancer types show intrinsic resistance to BRAFV600E specific inhibitors, mainly due to feedback activation of EGFR. This is treated with combined treatments that include BRAF and EGFR inhibitors to achieve encouraging preclinical, as well as clinical results [13].

Exploitation of TRAIL apoptotic properties for cancer therapy has provided encouraging results in the last decade. TRAIL induces apoptosis via interacting with its death receptors DR4 and DR5, which in turn results in death-inducing signaling complex (DISC) formation and caspase-8 processing [20]. Caspase-8 activation can then result in caspase-3 activation through the mitochondrial-independent pathway, and/or through the activation of Bid through the mitochondrial-dependent pathway [21]. Moreover, many studies have shown that TRAIL is more efficient in induction of cancer cell death in combined treatments [22]. In recent studies, 5-Fluorouracil displayed synergy with TRAIL in inducing apoptosis in mutant *KRAS* non-small cell lung carcinoma cells [23]; TRAIL-R2-specific antibodies and recombinant TRAIL can synergise to kill cancer cells [24].

Targeting BCL-2 anti-apoptotic complexes and pathways in cancer is a productive drug discovery and development field. The small molecule ABT-199, which antagonizes the activity of BCL-2, is one of the most promising examples being currently in clinical trials and shows activity in many lymphoid malignancies as a single agent and in combination with conventional chemotherapy agents [25, 26].

Apoptosis inhibition contributes to the survival and proliferation of tumors and plays an important role to current therapy resistance. Targeting apoptosis is therefore very promising for the development of new agents that may enhance current cancer therapies. Birinapant (TL32711), C_42_H_56_F_2_N_8_O_6_, is an antagonist of XIAP and cIAP1 with K_d_ value of 45 nM and <1 nM, respectively (K_d_ is the equilibrium constant involved in the dissociation of a compound into two or more compounds; the lower the K_d_ value the higher the affinity of the compound with the IAPs). Birinapant is a second-generation bivalent antagonist of IAP proteins that is currently undergoing clinical development for the treatment of cancer. It has been demonstrated, using a range of assays that evaluated cIAP1 stability and oligomeric state, that Birinapant stabilized the cIAP1-BUCR (BIR3-UBA-CARD-RING) dimer and promoted auto-ubiquitylation of cIAP1 in vitro, and this improved tolerability has allowed Birinapant to proceed into clinical studies [14]. The pro-apoptotic effects of Birinapant on caspase-3 activation were evaluated in mice bearing 38C13 B-cell lymphoma, HCT116 colon carcinoma or MDA-MB-231 breast adenocarcinoma tumours [15].

AT-406 (SM-406), C_32_H_43_N_5_O_4_, is a novel and orally active antagonist of multiple IAP proteins (binds to XIAP, cIAP1 and cIAP2). This is the first SMAC-mimetic registered for clinical trials in patients with advanced cancer. Limited anti-tumour activity may suggest development rather as adjunct treatment [16]. AT-406 acts as a strong radio sensitizer in human cervical cancer cells [17] and has demonstrated anti-ovarian cancer efficacy as a single agent and in combination with carboplatin [18]. In addition, AT-406 is highly effective in induction of apoptosis in xenograft tumours and is currently in phase I clinical trials for the treatment of of solid and hematological human tumors [19].

In this study, we investigate the effect of IAPs inhibition by recently developed SMAC-mimetics Birinapant and AT-406 in colorectal tumour cells, their cross-talk with the TRAIL-induced apoptotic pathway, BRAF and BCL-2 oncogenic pathways and the underlying mechanisms that can efficiently overcome tumour resistance to apoptosis. Efficient protocols of inhibition of IAPs activity and anti-apoptotic effect are presented by using Birinapant or AT-406 alone and in their combinations with either TRAIL or with other inhibitors of pro-survival pathways, like BRAF-MEK and BCL-2. Synergistic rational anticancer combined protocols are presented depending on the tumour cell background, like resistance to individual treatments, BRAF mutation or BCL-2 overexpression. These can be later further exploited in vivo, thus validating a precision medicine approach.

## Methods

### Cell lines

DLD-1, HCT116, SW620, HT29, RKO, Colo-205 human colon adenocarcinoma and Caco-2 colon intermediate adenoma cell lines were obtained from American Type Culture Collection (ATCC). All cell lines used in this study were grown in D-MEM medium supplemented with 10 % Fetal Bovine Serum (#10270, ThermoFisher Scientific, Wlatham, MA, USA, antibiotics (penicillin/streptomycin) and amino acids. Cells were treated with the SMAC-mimetics Debio1143 (or AT-406) and TL32711 (or Birinapant, catalog No. S7015, Shelleck Chemicals, Europe) that block the interaction of IAPS with caspases. Cells were also treated with the BRAFV600E inhibitor PLX-4720 (catalog No. S1152, Shelleck Chemicals, Europe), the BCL-2 inhibitor ABT-199 (GDC-0199) (catalog No. S8048, Shelleck Chemicals, Europe) and TRAIL SuperKiller cc-TRAIL (ALX-522-020) (Alexis Biochemicals, Laussane, Switzerland).

### Western blotting

Whole cell lysates were prepared with RIPA Buffer [50 mM Tris HCl pH: 8, 150 mM NaCl, 0.5 % sodium deoxycholate, 1 % NP-40, 10 % SDS]. Extracts were resolved on SDS-PAGE, and transferred to nitrocellulose membrane (Whatman, Scheicher & Schuell, Dassel, Germany). Membranes were incubated with the specific antibodies overnight at 4 °C, washed with TBS-Tween20 and incubated with the appropriate secondary antibody, for 1 h at room temperature. Antibodies were used against: XIAP (1:500-#610716, BD Biosciences, San Jose, CA, USA), cIAP-1 and cIAP-2 (1:750-/#sc-7943 and sc-7944, Santa Cruz Biotechnology, Heidelberg, Germany), DR4 and DR5 (1:5000-/#1139 and #2019, ProSci Incorporated, Poway, CA, USA),, FADD (1:1000-/#F36620, BD Biosciences, San Jose, California, USA), BID (1:500-/#sc-11423, Santa Cruz Biotechnology, Heidelberg, Germany), BAD (1:200-/#sc-941, Santa Cruz Biotechnology, Heidelberg, Germany), BAX (1:500-/#MS-711, ThermoFisher Scientific, Waltham, MA, USA), BCL-2 (1:200-/#sc-7382, Santa Cruz Biotechnology, Heidelberg, Germany), Tubulin (1:2000-#sc-8035, Santa Cruz Biotechnology, Heidelberg, Germany), Cleaved Caspase-3 (1:500-/#9661, Cell Signaling Technology, Danvers, MA, USA), Total Caspase-3 (1:1000-/#9662, Cell Signaling Technology, Danvers, MA, USA), PARP-1 (sc-7150, Santa Cruz Biotechnology, Heidelberg, Germany), RIP-1 (1:2000-/#610458, BD Biosciences, San Jose, CA, USA), RIP-3 (1:2000-/#NBP2-24588, Novus Biologicals, Littleton, CO, USA). Secondary antibodies were: Goat anti-mouse IgG-HRP Santa Cruz Biotechnology, sc-2005; a-rabbit IgG-HRP, Jackson, 111-035-003. All the antibodies were diluted in 5 % skim milk”. Antibody signal was obtained with the enhanced chemiluminescence plus Western blotting detection system (Amersham Biosciences, Uppsala, Sweden) after exposure to Kodak Super RX film. Values were measured using the Image-Quant software (Amersham Biosciences) and protein levels were normalized against housekeeping proteins (tubulin/GAPDH). Experiments were independently repeated three times and standard deviation is presented.

### Two dimensional culture

For the 2D culture experiments, cells (5×10^3^ cells/well) were grown on cover slips in 24-well plates in medium, at 37 °C. Photographs of the 2D cultures were taken under light and confocal microscope after the selected treatments and appropriate staining.

The nuclei were stained with Hoechst No. 33342 (catalog No. 62249, ThermoFisher Scientific, Waltham, MA, USA) for apoptosis detection; the cleaved caspase-3, a marker of apoptosis, was detected with cleaved caspase-3 antibody (#9661, Cell Signaling Technology, Danvers, MA, USA) and a fluorescent secondary antibody (Alexa Fluor 488, #A11008, ThermoFisher Scientific, Waltham, MA, USA)

### Three dimensional culture

For three-dimensional culture Matrigel (catalog No. 356234, BD Biosciences, San Jose, CA, USA) was used. Poly-lysine pre-coated cover slips were put in 24-well plate. Matrigel was diluted with cold complete DMEM medium to a final concentration of 50 %, 200 μl were deposited on each well containing cover slips and the plate was incubated at 37 °C for 15’. 1-1.5×10^3^ cells was diluted in 100 μL of cold DMEM and mixed with 100 μL of 100 % Matrigel. The resulting 200 μL were added to the wells with pre-warmed Matrigel. Plate was incubated in a humidified atmosphere at 37 °C with 5 % CO_2_ for 13–15 days. Photographs of the 3D cultures were taken under light and confocal microscope after the selected treatments and appropriate staining. The nuclei were stained with Hoechst No. 33342 (catalog No. 62249, ThermoFisher Scientific, Waltham, MA, USA) for apoptosis detection; the cleaved caspase-3, a marker of apoptosis, was detected with cleaved caspase-3 antibody (#9661, Cell Signaling Technology, Danvers, MA, USA) and a fluorescent secondary antibody (Alexa Fluor 488, #A11008, ThermoFisher Scientific, Waltham, MA, USA)

### Cell viability assay

For growth studies the sulforhodamine B (SRB) (S1402-5G, Sigma-Aldrich, Taufkirchen Germany) assay was used. Firstly, tumour cells (5**×**10^3^ cells/well) were seeded into 96-well micro titer plates and were allowed to attach overnight. Thereafter, the cell number in treated versus control wells was estimated after treatment with 10 % trichloroacetic acid and staining with 0.4 % SRB in 1 % acetic acid. The percentage of viable cell was plotted each time. SD was used for error bar generation. For the calculation of combined drug effects, the median effect analysis was used [27, 28]. Synergism was determined using the method previously described based on the Bliss Independence Model [28, 29].

### Cell migration assay

Cells were trypsinised, washed with medium containing 1 % FBS, and counted. 10^5^ cells were plated into upper chamber of an 8 μm-pore Transwell filter (#3422, Corning, NY, USA), mounted in a 24-well dish containing 10 % FBS medium. Filters were pre-coated with Fibronectin (f1141, Sigma-Aldrich, Taufkirchen, Germany). Cells were allowed to migrate at 37 ^o^C, 5 % CO_2_ for 36–40 h, fixed with methanol and stained with 0.1 % w/v crystal violet. Underside of filters was observed with 40× objective and migrating cells were determined in ten randomly selected fields for each well. Experiments were performed in duplicate and repeated twice. For migration assay coupled with SMAC-mimetic, cells were treated with Birinapant for 24 or 48 h and then seeded on transwell in the presence of Birinapant and incubated at 37 °C for another 24 h.

### RNA extraction, reverse transcription and real time-PCR

Total RNA isolation from cultured cells as well as cancer specimens was performed using the Trizol reagent (Invitrogen, Karlsruhe, Germany). Reverse transcription was carried out from 3.0 μg of purified RNA using the SuperScript Reverse Transcriptase (Invitrogen, Karlsruhe, Germany) following the manufacturer’s instructions. Real-time quantification at the mRNA level was carried out in 96-well PCR plates using a Bio-Rad iCycler and the iQ5 Multicolor real-Time PCR detection system (Bio-Rad, Hercules, CA, USA). Each reaction contained 1× iQ SYBR Green Supermix (Bio-Rad, Hercules CA, USA) and 150 nmol/L of each primer. All genes were tested in duplicates. Results were analyzed on the iCycler software. Values were normalized to GAPDH. Primers used were the following:

- GAPDH: 5’-GAA GGT GAA GGT CGG AGT (FW)
  5’-CAT GGG TGG AAT CAT ATT GGA (RV)
- cIAP-1: 5’-TGT TGT CA ACTT CAG ATA CCA CTG G-3’ (FW)
  5’-CAT CAT GAC AGC ATC TTC TGA AGA-3’ (RV)
- cIAP-2: 5’-TCC GTC AAG TTC AAG CCA GTT-3’ (FW)
  5’-TCT CCT GGG CTG TCT GAT GTG-3’ (RV)
- XIAP: 5’-GAC AGT ATG CAA GAT GAG TCA AGT CA-3’ (FW)
  5’-GCA AAG CTT CTC CTC TTG GAG-3’ (RV)
- BIRC5: 5’-GCA CGG TGG CTT ACG CCT G-3’ (FW)
  5’-AAC CGG ACG AAT GCT TTT TAT CC-3’ (RV)
- DR4: 5’-TCC AGC AAA TGG TGC TGA C-3’ (FW)
  5’-GAG TCA AGG GGC ACG ATG TT-3’ (RV)
- DR5: 5’-CCA GCA AAT GAA GGT GAT CC-3’ (FW)
  5’-GCA CCA AGT CTG CAA AGT CA-3’ (RV)

## Results

### XIAP, DR4 and DR5 are overexpressed at the mRNA and protein levels in all adenocarcinoma cell lines. High levels of BCL-2 protein expressed in RKO cell line

Relative RNA levels of cIAP-1, cIAP-2, XIAP, SURVIVIN, DR4 and DR5 genes were evaluated by Real Time PCR analysis in seven colon cancer cell lines. Caco-2 is an intermediate adenoma cell line and was used as control. The other six cell lines are colorectal adenocarcinoma cell lines: DLD-1 and HCT116 bear *KRAS* and *PI3KCA* mutations, SW620 bears *KRAS* mutation, HT29 and RKO bear *BRAF* and *PI3KCA* mutations and Colo205 cell line bears *BRAF* mutation.

**Fig. 1:**
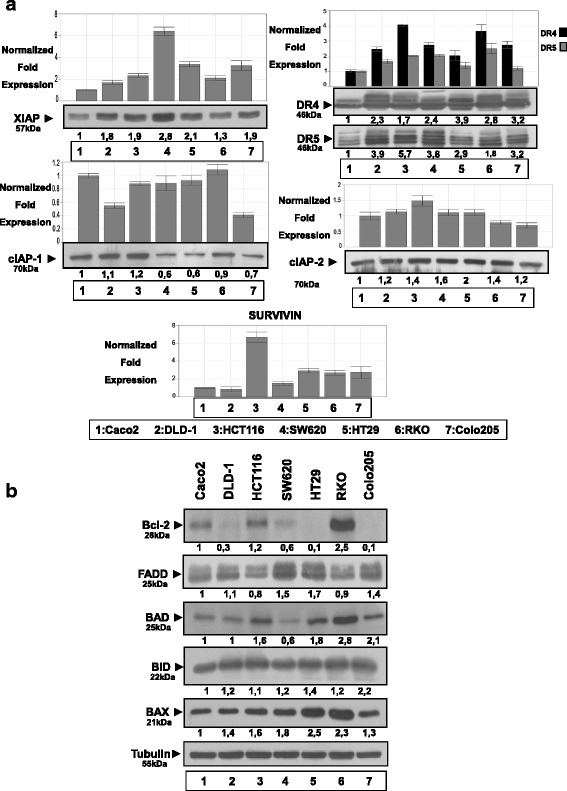
Overexpression of IAP members, TRAIL receptors DR4, DR5 and BCL-2 members in colorectal adenocarcinoma cell lines: DLD-1 (lane 2), HCT116 (lane 3), SW620 (lane 4), HT29 (lane 5), RKO (lane 6) and Colo-205 (lane 7), compared to Caco2, an intermediate colorectal adenoma cell line (lane 1). **a**: Relative RNA and protein levels of cIAP-1, cIAP-2, XIAP, SURVIVIN, DR4 and DR5 genes were evaluated by Real Time PCR and W.B analysis respectively in colon adenocarcinoma cell lines. The analysis was performed in triplicates and the ± SD is shown. Columns indicate relative RNA levels normalized to glyceraldehyde 3-phosphate dehydrogenase (GAPDH). Proteins are quantified against α-Tubulin. **b**: Protein levels of other (anti)apoptotic factors in colon adenocarcinoma cell lines by W.B. Data are representative for three independent experiments

XIAP mRNA is overexpressed in all adenocarcinoma cell lines as compared to Caco-2, mostly in SW620 (over 6-fold) (Fig. 1a, lane 4) and in HT29 and Colo205 (up to 4-fold) (Fig. 1a, lanes 5 and 7, respectively). Protein levels of XIAP were also found up-regulated in all cell lines compared to Caco-2 and especially in SW620 (2.8-fold) (Fig. 1a, lane 4), in HT29 (2.1-fold), in HCT116 and Colo205 (1.9-fold) and in DLD-1 (1.8-fold) (Fig. 1a, lanes 5, 3, 8 and 2 respectively).

TRAIL receptors expression was evaluated by Q-PCR and western blot analysis: DR4 mRNA is mostly over-expressed in HCT116 and RKO (4-fold), as well as in SW620 and Colo205 (up to 3-fold) compared to Caco-2 respectively (Fig. 1a. lanes 3, 6, 4, and 7). On the other hand, protein levels of DR4 were found over-expressed in all cell lines. In HT29 cell line by 3.9 fold, in Colo205 by 3.2 fold, in RKO by 2.8 fold, in SW620 by 2.4 and in DLD-1 by 2.3 fold compared to Caco-2 intermediate adenoma cell line respectively (Fig. 1a, lanes 5, 7, 6, 4, 2). DR5 mRNA was also found over-expressed in all cell lines as compared to Caco-2, mostly in RKO (2.5-fold), as well as in HCT116 and SW620 (2-fold) cell lines compared to Caco-2 (Fig. 1a, lanes 6, 3, 4 respectively). Protein levels of DR5 were detected remarkably up-regulated in all cell lines compared to Caco-2: from 5.7-fold in HCT116 to 1.8-fold in RKO, as compared to Caco-2 cell line (Fig. 1a).

mRNA levels of cIAP-1 and cIAP-2 have no significant differences between Caco-2 and adenocarcinoma cell lines, while cIAP-2 protein was over-expressed in all cell lines compared to Caco-2 (Fig. 1a). mRNA level of SURVIVIN is overexpressed in all cell lines but mostly in HCT116 as compared to Caco-2 (Fig. 1a). On the other hand, most of examined BCL-2 family members involved in the intrinsic death pathway were found over-expressed in almost all adenocarcinoma cell lines utilized: BCL-2 protein in RKO cell line being the most upregulated, other overexpressed factors of this family are BAK and BAX (Fig. 1b).

### Treatment with SMAC-mimetics Birinapant and AT-406 resulted in a decrease of cell viability and appearance of apoptotic characteristics in colorectal adenocarcinoma cells

**Fig. 2:**
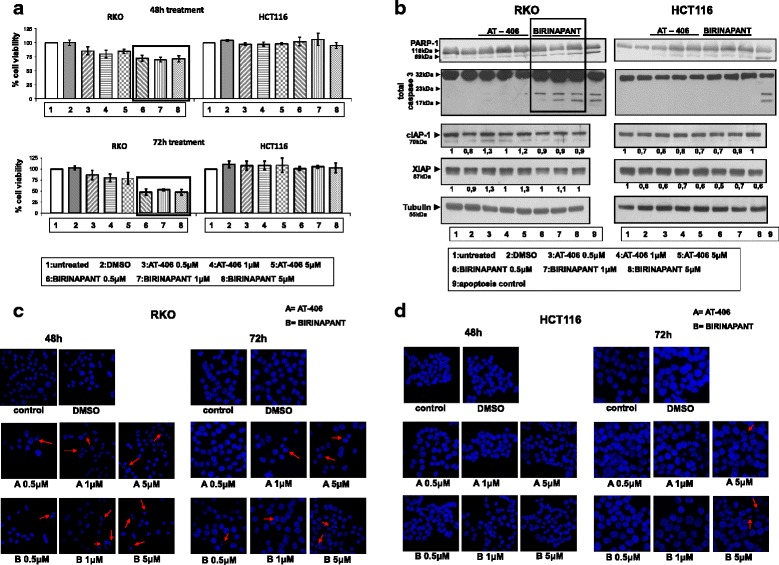
Birinapant treatment results in reduced cell viability and appearance of apoptosis in selected colorectal adenocarcinoma cells. **a**: Cell viability of cell lines after treatment with SMAC-mimetics. Cells were either left untreated (ctr = control) or treated with 0.5 μM, 1 mM or 5 mM AT-406 and Birinapant for 48 h (1) and 72 h (2) and the % percentage cell viability was measured by SRB. Average of three independent experiments are presented as fold change of the absorbance of treated/untreated cells for each condition. Columns = % percentage of cell viability, bars = SD. **b**: Protein levels of XIAP, cIAP-1, PARP-1 and total caspase-3 in RKO and HCT116 were analysed by W.B. after treatment with 0.5, 1 and 5 μM SMAC-mimetics AT-406 (lanes 3–5) and Birinapant (lanes 6–8) for 48 and 72 h. Untreated (lane 1) or treated with DMSO cells (lane 2) were used as control. Proteins are quantified against α-Tubulin. Data are representative for three independent experiments. **c**-**d**: Confocal microscope images and Hoechst staining of RKO (2C) and HCT116 (2D) cell lines two-dimensional culture, after treatment with SMAC-mimetics AT-406 (A) and Birinapant (B). Crescent nuclei of RKO cells present after cell treatments are shown by arrows. Confocal microscope images were taken after treatment with SMAC-mimetics AT-406 and Birinapant in RKO (2c) and HCT116 (2d) 48 and 72 h. The nuclei were detected with HOECHST staining. Representative images are presented

The effect of IAP inhibitors on colorectal adenocarcinoma cell lines was examined by cell treatments with SMAC-mimetics Birinapant or AT-406 in RKO and HCT116 colorectal adenocarcinoma cell lines. Up to 50 % decrease of cell viability in RKO cell line after 72 h treatment with the SMAC-mimetic Birinapant in several doses was observed (Fig. 2a, lower panel), while shorter treatment (48 h) resulted in 25–30 % reduction of cell viability of RKO cells (Fig. 2a, upper panel).

Following the above findings regarding reduction of cell viability after Birinapant treatment, the effect of SMAC-mimetics treatments on apoptosis was tested. After 72 h treatment of RKO cells with 0.5–5 μM Birinapant, clear signs of apoptosis were detected, as shown by caspase-3 and PARP-1 cleavage (Fig. 2b, lanes 6, 7, 8) and by detection of fragmented nuclei in RKO cells (Fig. 2c, lower panel). On the other hand, AT-406 treatment in RKO cells resulted in a lower reduction of cell viability (15–20 %), as compared to Birinapant, while little effect on apoptotic markers like PARP-1 cleavage, caspase-3 cleavage (Fig. 2b, lanes 3–5), was detected, further confirmed by small number of crescent nuclei (Fig. 2c, middle panel). Parallel treatments of Birinapant and AT-406 in HCT116 cell line, resulted in no significant effects on either cell viability (Fig. 2a) or apoptotic markers (Fig. 2b and d), respectively

### Birinapant treatment results in the reduction of cell migration properties of RKO cell line

**Fig. 3:**
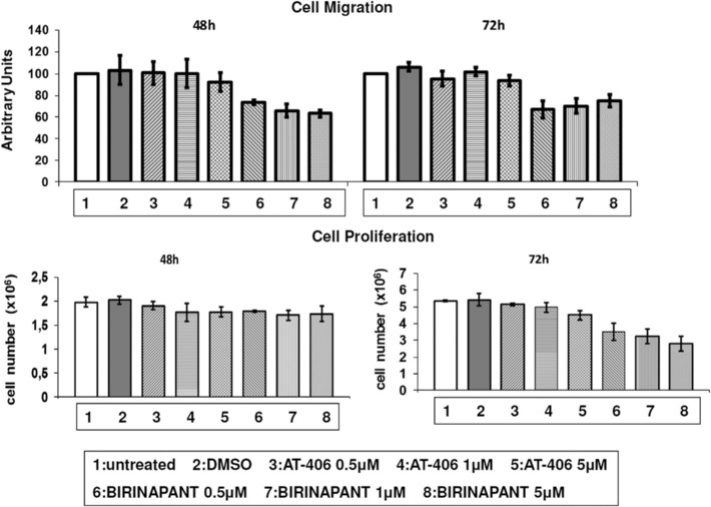
Birinapant treatment reduces RKO tumour cell migrative capacity. Upper panel: Migration ability of RKO cell lines treated with SMAC-mimetics AT-406 and Birinapant compared to the untreated cells. Cells were treated with AT-406 and Birinapant for 24 or 48 h and allowed to migrate. The values are the average of two independent experiments. Lower panel: Cell proliferation of cell line RKO co-treated with SMAC-mimetics AT-406 and Birinapant for 48 and 72 h. The values are the average of two independent experiments. Columns = number of cells/well (of 6-well plate)

Since IAPs were shown to be involved in cell migration [1], their potential effect of their inhibition on cell migration properties in colorectal adenocarcinoma cells was tested (Fig. 3, upper panel). Indeed, after 48 or 72 h treatment with 0.5 μM, 1 μM and 5 μM SMAC-mimetics Birinapant or AT-406, less migrative cells were detected, as compared to the untreated cells (Ctr), which was more evident with Birinapant treatments in all doses (Fig. 3, upper panel). Cell numbers after the corresponding treatments are presented in Fig. 3, lower panel. As indicated here, Birinapant and to a less extent AT-406, have shown potency towards colorectal adenocarcinoma cell death and inhibition of cancer cell migration properties.

The mild treatment effects of the tested SMAC-mimetics presented in the first part of this study, proposed for rational combined treatment protocols in resistant cell lines to overcome resistance. Co-treatments presented in the following second part of the study include rational combinations of SMAC mimetics with a) oncogenic BRAFV600E inhibitors, also not efficient as mono-treatments, b) other therapeutics targeting apoptosis, like TRAIL and c) BCL-2 inhibitors, in cells presenting high BCL-2 expression.

### SMAC-mimetics synergise with BRAFV600E inhibitor towards efficient antitumour treatments in 3D cultures of colorectal adenocarcinoma cells

**Fig. 4:**
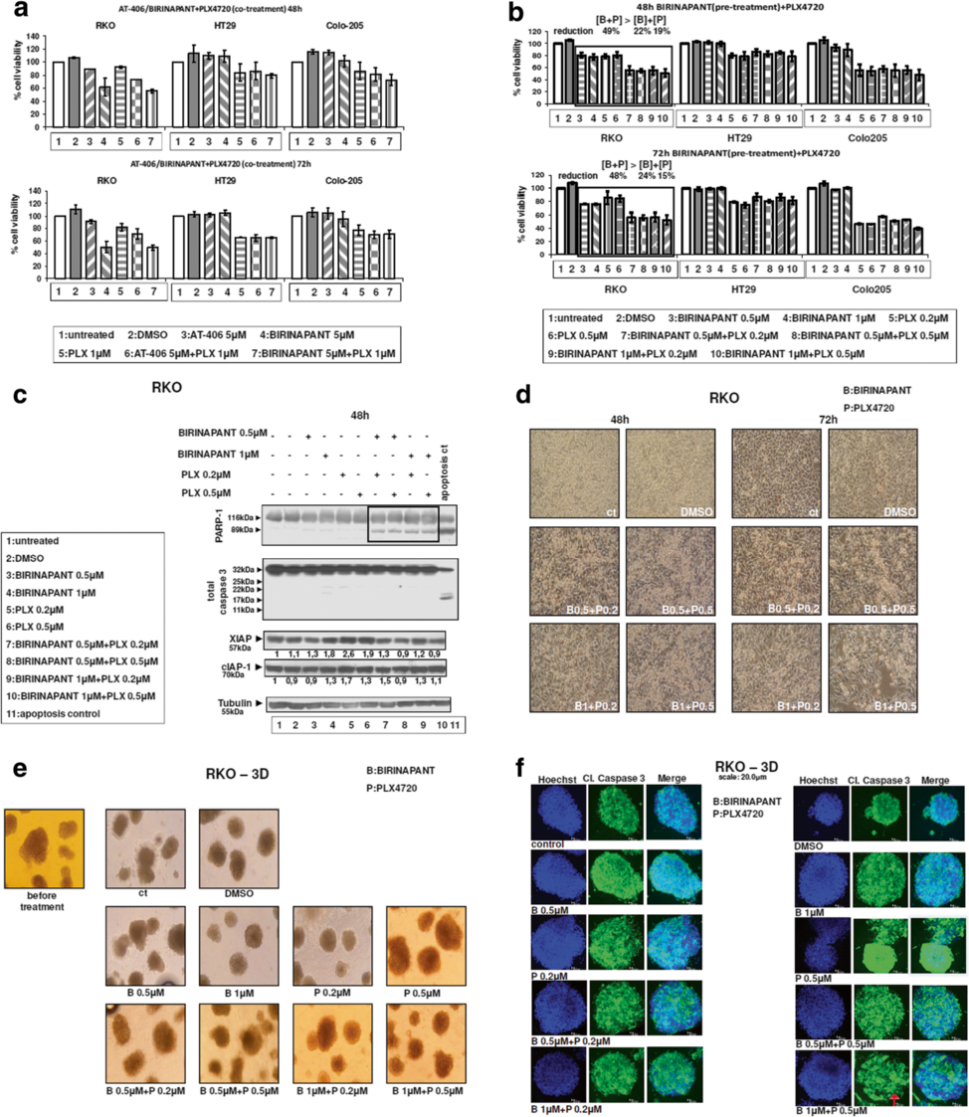
Pre-treatment with Birinapant and then co-treatment with Birinapant/ BRAF inhibitor PLX4720 synergistically induce apoptosis of colorectal adenocarcinoma cells in 2D and 3D. **a**: Cell viability after co-treatment with the SMAC-mimetics Birinapant or AT-406 in combination with the BRAF inhibitor PLX4720. Cells were either left untreated (ctr = control) or treated with 5 μM Birinapant, 5 μM AT-406 and 1 μM PLX4720 and their combinations for 48 and 72 h. The average of three independent experiments is presented as fold change of the absorbance of treated/untreated cells, for each condition. Columns = % percentage of cell viability, bars = SD. **b**: Cell viability after pre-treatment with the SMAC-mimetic Birinapant and then co-treatment with BRAF inhibitor PLX4720 and Birinapant. Cells were either left untreated (ctr = control) or treated with 0.5 or 1 μM Birinapant and 0.2 or 0.5 μM PLX4720. For the pre-treatment testing, cells were first incubated for 24 h with either 0.5 or 1 μM of Birinapant and then co-treated with 0.2 and 0.5 μM PLX4720 for another 24 or 48 h. **c**: Protein levels of PARP-1, total Caspase-3, XIAP, cIAP-1 and p-ERK1/2 in RKO by W.B., after pre-treatment with 0.5 or 1 μM Birinapant and then co-treatment with 0.2 or 0.5 μM PLX4720. Untreated cells were used as control. Proteins are quantified against α-Tubulin. Data are representative for three independent experiments. **d**: Light microscopy images from RKO culture after pre-treatment with Birinapant and then co-treatment with PLX4720. Detached cells are shown in supernatant of co-treated group. Several images were taken from untreated and treated with Birinapant (0.5 or 1 μM) and PLX4720 (0.2 or 0.5 μM) RKO cells while been cultured in 6-well plates for 48 and 72 h. Representative images are presented. **e**: Light microscopy of three-dimensional culture of RKO cells after co-treatment with 0.5 μM, 1 μM Birinapant and 0.2 μM, 0.5 μM PLX4720 and their combination in 3D culture for 6 d. Representative images. **f**: Confocal microscope images were taken after co-treatment with 0.5 μM, 1 μM Birinapant and 0.2 μM, 0.5 μM PLX4720 and combinations in 3D cultures for 6 days. The nuclei were detected with HOECHST staining (blue color), cleaved Caspase-3 (green color). Representative images. Scale bar: 20 μm

RKO colon adenocarcinoma cells were partially resistant to SMAC-mimetics Birinapant and AT-406, as shown in previous experiments (Fig. 2a). Moreover, it has been shown in many studies and clinical trials that colorectal tumours and cell lines are resistant to specific BRAFV600E inhibitors PLX4720 and PLX4032 (Vemurafenib). Therefore, the potential synergy of Birinapant or AT-406 with PLX4720 in killing colorectal cancer cells was tested. Co-treatments of Birinapant with PLX4720 did not improve the efficiency of individual mono-treatments (Fig. 4a). Notably, a synergistic effect in the reduction of cell viability of RKO cell line was observed after applying a treatment protocol involving Birinapant pre-treatment and then combined treatment of Birinapant with PLX4720 for 48 and 72 h: as a result 49 and 48 % synergistic reduction of cell viability in RKO cells respectively was reported, 8 and 9 % more than the sum of the mono-treatments of Birinapant and PLX4720 at the same time points respectively (Fig. 4b). The above combined protocol using Birinapant pre-treatment, followed by Birinapant-PLX4720 co-treatment have caused the appearance of apoptotic markers in RKO cells, like PARP-1 cleavage after 48 h (Fig. 4c, lanes 7–10). Further evidence is provided by light microscopy images, where the detached RKO cells after this combined treatment are shown, especially after 72 h combined treatment (Fig. 4d, right panel). Ex vivo conditions which can mimic the in vivo environment can be provided by 3D culture treatments. In 3D culture, a disorganization of some of the tumour colonies was detected (Fig. 4e, lower panel) and higher staining of cleaved caspase-3 was observed (Fig. 4f) after the combined treatment of Birinapant and PLX4720 for 6 days. A minor effect on cell properties using these two assays was shown during 3D culture mono-treatments (Fig. 4e and f). Finally, the combined treatment of the other SMAC-mimetic AT-406 with PLX4720 did not provide any advantage in the reduction of RKO tumour cell viability, as compared to mono-treatments (Additional file 1: Figure S3).

### Treatment with SMAC-mimetics Birinapant and AT-406 can sensitize resistant-to-TRAIL cancer cells to apoptosis in 2D and 3D cell cultures

**Fig. 5:**
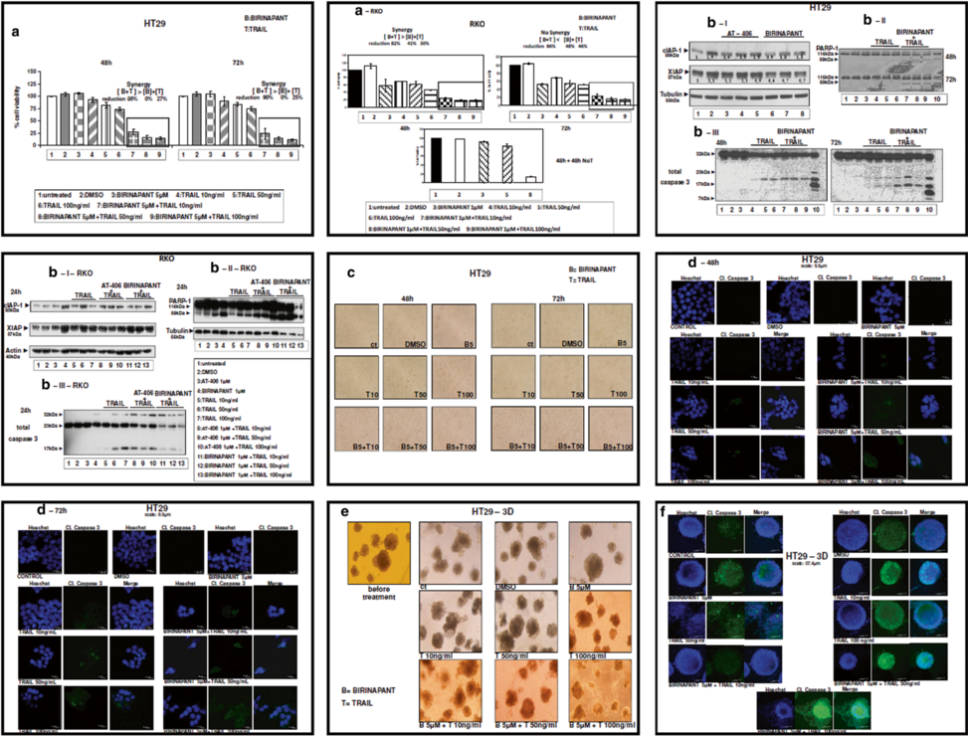
Co-treatment of Birinapant with TRAIL can synergistically increase their efficiency and induce apoptosis in colorectal adenocarcinoma cells in 2D and 3D. 5a: Cell viability of cell line HT29 after co-treatment with the SMAC-mimetic Birinapant and the apoptotic agent TRAIL. Cells were either left untreated (ctr = control) or treated with Birinapant, TRAIL and their combination for 48 and 72 h and the % percentage cell viability was measured. The average of three independent experiments is presented. Columns = % percentage of cell viability, bars = SD. 5a-RKO: Respectively for cell line RKO (upper panel). To check for the reversibility of treatment effects, RKO cells were either left untreated (ctr = control), treated with either Birinapant, TRAIL or their combination for 48 h. Cells were then incubated with additional 48 h without treatments (NoT) and the % percentage cell viability was measured. The average of three independent experiments is presented. 5b-I. Protein levels of cIAP-1 and XIAP after 0.5, 1 and 5 μM AT-406 (lanes 3–5) or Birinapant (lanes 6–8) treatments respectively for 48 h, compared to control lanes 1 (no treatment) or 2 (DMSO treatment). 5b-II and 5b-III. Protein levels of PARP-1 and total Caspase-3 in HT29 cell line after co-treatment with SMAC-mimetic Birinapant and TRAIL for 48 and 72 h. Cells were either left untreated (lane 1) or treated with DMSO (lane 2), with 5 μM Birinapant (lane 3), with 10, 50 and 100 ng/mL TRAIL (lanes 4–6), or with 5 μM Birinapant and combination with TRAIL (lanes 7–9) for 48 and 72 h. Sensitive cell line HCT116 treated with TRAIL is used as positive control (lane 10). Using W. B., protein levels of PARP-1 (Figure 5b-II) and of total Caspase 3 (Figure 5b-III) were analyzed after the corresponding treatments. Data are representative for three independent experiments. 5b-I-RKO: Protein levels of cIAP-1 and XIAP after mono-treatments (lanes 3 and 4 for AT-406 and Birinapant respectively and lanes 5–7 for TRAIL 10, 50 and 100 ng/mL respectively) and co-treatments (lanes 8–13 as shown) for 24 h. Untreated (lane 1) and treated with DMSO (lane 2), cells are also presented. 5b-II-RKO and 5b-III-RKO: Protein levels of PARP-1 and total Caspase-3 after respective mono-treatments and co-treatments (same lines as 5-I-RKO). Data are representative of three independent experiments. 5C: Light microscopy images from HT29 culture after combined treatment with Birinapant and TRAIL. Detached (apoptotic) cells are shown in supernatant of co-treated groups for 48 and 72 h. Representative images. 5d: Confocal microscope images were taken after co-treatment with Birinapant, TRAIL and their combination. Nuclei were detected with HOECHST staining (blue color), cleaved Caspase-3 (green color). Representative images.. Scale bar: 9.9 μm. 5e. Light microscopy of three-dimensional culture of HT29 cells after co-treatment with Birinapant, TRAIL and their combinations in 3D for 6 days. Representative images. 5f: Confocal microscope images were taken after co-treatment with Birinapant, TRAIL and their combinations in 3D culture for 6 days. The nuclei were detected with HOECHST staining (blue color), cleaved Caspase-3 (green color). Representative images. Scale bar: 57.4 μm

As HT29 (mainly) and RKO cell lines appear partially resistant to TRAIL [27, 28] and has shown little response to Birinapant, we decided to check if the combined treatment of these two drugs would be more efficient in killing HT29 and/or RKO cells. Combined treatments of Birinapant and TRAIL for 48 or 72 h resulted in up to 90 % significant reduction of cell viability in HT29, a cell line that appears partially resistant in TRAIL-induced apoptosis (Fig. 5a). Combined treatments of Birinapant and TRAIL in RKO resulted in reduction of cell viability up to more than 85 % compared to the untreated cells, or those which were mono-treated, both at 48 or 72 h (Fig. 5a-RKO, upper panel). Notably, under these conditions, the effect of combined Birinapant and TRAIL treatments on RKO cell viability was irreversible after additional cell incubation without drug, which was not the case in the mono-treatments (Fig. 5a-RKO, lower panel, lane 8 compared to 1, 2, 3 and 5). Combined treatments of Birinapant with TRAIL not only decreased cell viability in both cell lines, but resulted in clear appearance of apoptotic markers, like PARP-1 and caspase-3 cleavage (Fig. 5b-II and 5b-III, lanes 7–9 and Fig. 5b-III- RKO, lanes 11–13) as compared to non-treated cells (lanes 1, 2), or mono-treated cells (lanes 3 and 4–6). HCT116 cells treated with TRAIL were used as positive control of apoptosis (lane 10). Efficiency of Birinapant combined treatments can be due to the effect of 0.5, 1 and 5 mM Birinapant treatment on the reduction of XIAP protein levels after 48 h (Fig. 5b-I, lanes 6–8 respectively compared to control lane 1), although the effect on cell viability are not optimal under the same conditions. In other words, Birinapant treatment may prime HT29 cells to TRAIL induced cell death by reducing XIAP expression levels. In RKO cell line there is no evident reduction of XIAP levels after 24 h (Fig. 5b-I-RKO) or 48 h (data not shown). Additional evidence for the TRAIL-Birinapant combined treatment efficiency in HT29 cells was provided by light microscopy images, showing detached HT29 cells after 48 or 72 h TRAIL-Birinapant combined treatments (Fig. 5c, left or right panel respectively), while confocal microscopy images indicated the fragmented (apoptotic) nuclei and cleaved caspase-3 distribution in a number of treated tumour cells (Fig. 5d). Lower concentration of Birinapant (0.5 μM and 1 μM) in combination with TRAIL did not show any synergistic effect on HT29 tumour cell viability (Additional file 2: Figre S2a and S2b), but a higher concentration of Birinapant (10 μM) presented similar synergistic effect in combination with TRAIL, as with the lower concentrations 0.5–5 μM (Additional file 2: Figure S2c). As shown here, the effect of combined Birinapant and TRAIL treatments on RKO cell viability was synergistic and irreversible.

In order to provide further evidence of Birinapant-TRAIL efficiency, co-treatment protocols were tested in models involving tumour microenvironment, like 3D tumour cell cultures: a significant reduction of tumour size and disorganization (Fig. 5e) and higher staining of cleaved caspase-3, as shown in confocal microscopy images (Fig. 5f) was detected after the combined treatment of Birinapant and TRAIL for 6 days. Effects after mono-treatments of individual drugs were also detected.

**Fig. 6:**
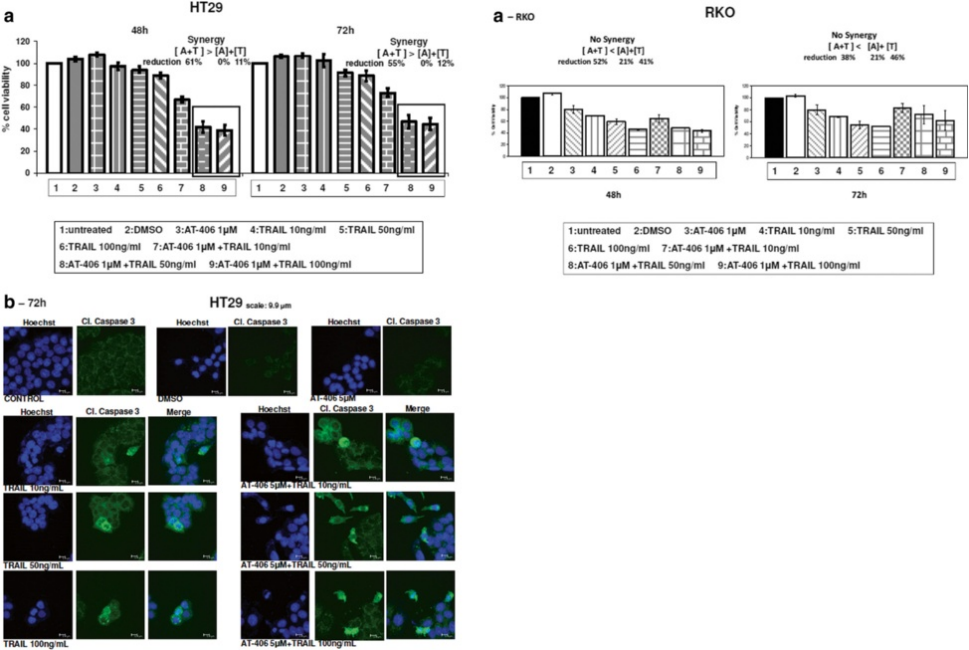
SMAC-mimetic AT-406 and TRAIL synergistically kill resistant tumour cells. 6a Cell viability of HT29 cell line after combined treatment with SMAC-mimetic AT-406 and the apoptotic agent TRAIL. Cells were either left untreated (ctr = control) or treated with AT-406 combined with TRAIL for 48 and 72 h and the % percentage cell viability was measured by SRB staining. The values are the average of three independent experiments and are presented as fold change of the absorbance of treated/untreated cells, for each condition. Columns = % percentage of cell viability, bars = SD. 6a-RKO: Respectively for cell line RKO. 6b. Confocal microscope images were taken after co-treatment with AT-406, TRAIL and their combinations for 48 and 72 h. The nuclei were detected with HOECHST staining (blue color), cleaved Caspase-3 (green color). Representative images. Scale bar: 9.9 μM

Furthermore, the efficiency of combined treatment with the SMAC-mimetic AT-406 and TRAIL in killing HT29 and RKO cells was tested. Similar synergistic effect is shown after AT-406 and TRAIL combined treatment of for 48 or 72 h in HT29 but not in RKO cells. A reduction up to 60 % of tumour cell viability was detected (Fig. 6a). The combined treatment with AT-406 and TRAIL resulted in cleavage of caspase-3 after 72 h treatment, in HT29 cells, as shown by confocal microscopy images (Fig. 6b).

**Fig. 7:**
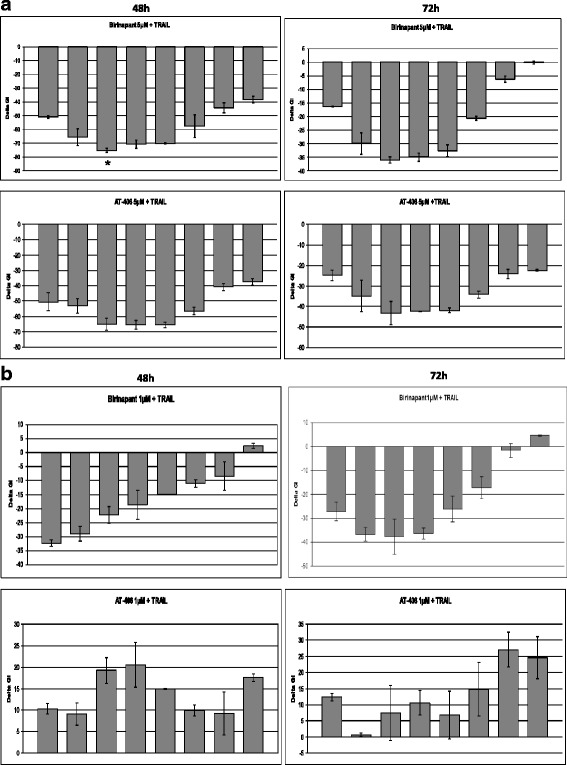
SMAC-mimetics AT-406 and Birinapant show synergistic effects when co-treated with TRAIL in HT29 and RKO. 7a-HT29: Synergy graphs for HT29 cell line treated for 48 and 72 h with AT-406 or Birinapant fixed dose (5 μM) and increasing concentrations of TRAIL (from left to right: 100, 50, 25, 12,5, 6,25, 3,125, 1,5625 and 0,78125 ng/mL). 7b-RKO: Respectively for RKO cell line with fixed dose of SMAC-mimetics 1 μM

The synergistic effects first presented above were also formally validated (Fig. 7) using the equation DeltaGI = GI_TRAIL_ + GI_Birinapant_(or GI_AT-406_)-GI_combination_, based on the Bliss Independence Model. In this model, values below zero indicate synergism. The cells were mono-treated with a fixed dose of Birinapant or AT-406 (5 μM in HT29 and 1 μM in RKO, because of their differential sensitization to SMAC-mimetics) and increasing concentrations of TRAIL (100 to 0,78125 ng/mL), as well as co-treated with the fixed dose of Birinapant and different TRAIL concentrations for 48 and 72 h. Synergistic effects in HT29 cells clearly appeared in treatments involving either of the two SMAC-mimetics at 48 and 72 h (Fig. 7a). The best synergistic effect was delivered by the combination of 5 μM Birinapant and 3.125 ng/mL TRAIL (*) which is up to -75 Delta GI. In RKO cell line instead, only Birinapant treatments show synergistic effects when combined with specific TRAIL concentrations (Fig. 7b).

### ABT-199, a specific inhibitor of BCL-2, reduces cell viability and has synergistic effect with SMAC-mimetics Birinapant and AT-406 in RKO tumour cells

**Fig. 8:**
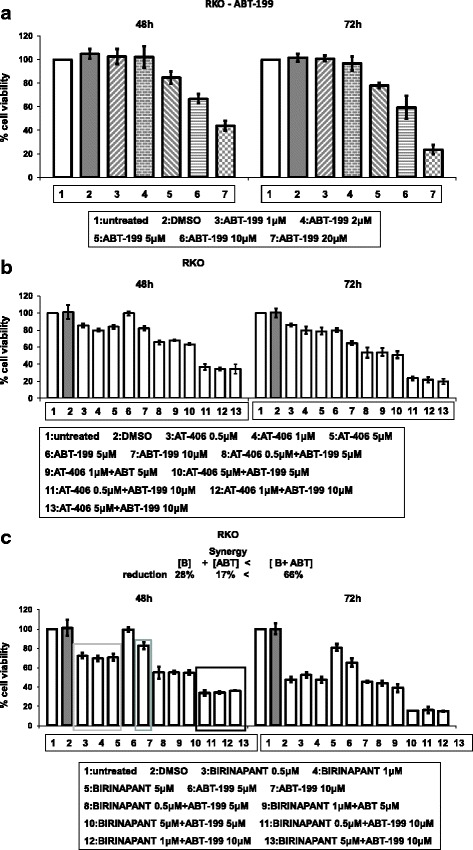
BCL-2 inhibitor ABT-199 can efficiently cause apoptosis when combined with SMAC-mimetics Birinapant and AT-406. **a**: Cell viability of RKO after treatment with ABT-199, a specific inhibitor of BCL-2. Cells were either left untreated (ctr = control) or treated with different concentrations of ABT-199 for 48 and 72 h and the % percentage cell viability was measured by SRB. The values are the average of three independent experiments and are presented as fold change of the absorbance of treated/untreated cells, for each condition. Columns = % percentage of cell viability, bars = SD. **b**: Cell viability of cell line RKO after combined treatment with the SMAC-mimetic AT-406 and ABT-199. Cells were either left untreated (ctr = control) or treated with AT-406 combined with ABT-199 for 48 and 72 h and the % percentage cell viability was measured by SRB. **c**: Cell viability of cell line RKO after combined treatment with the SMAC-mimetic Birinapant and ABT-199. Cells were either left untreated (ctr = control) or treated with Birinapant and ABT-199 combination for 48 and 72 h. The values are the average of three independent experiments and are presented as fold change of the absorbance of treated/untreated cells, for each condition. Columns = % percentage of cell viability, bars = SD

A notable overexpression of BCL-2 in RKO cell line is shown in Fig. 1c, therefore a specific BCL-2 inhibitor ABT-199 was used to test the importance of BCL-2 over-expression in RKO cell line viability and aggressiveness. ABT-199 treatment for 48 and 72 h resulted in reduction of RKO tumour cell viability, up to 60 and 80 % respectively, in concentrations 5–20 μM (Fig. 8a). Since RKO tumour cells appeared partially resistant to ABT-199 treatments, combined treatments of ABT-199 with SMAC-mimetics AT-406 or Birinapant were tested for potential improved efficacy. The co-treatment protocol of ABT-199 with AT-406 resulted in an enhanced effect on RKO tumour cell viability after 48 and 72 h, as compared to mono-treatments under the same conditions (Fig. 8b). Most notably, combined treatments of ABT-199 with Birinapant indicated a synergistic effect on RKO cell viability after 48 h treatment, as shown in Fig. 8c. Thus, in tumour cells with high BCL-2 expression, targeting apoptosis by combined protocols of BCL-2 with IAP inhibitors can provide efficient anti-cancer treatments in a synergistic manner.

## Discussion

The present study investigates the efficiency of SMAC-mimetics Birinapant and AT-406 in a number of CRC cell lines. Oncogenic and apoptotic pathways are exploited towards establishing novel as well as efficient anti-cancer treatment protocols, which involve either Birinapant or AT-406 as single agents or their rational combinations with TRAIL, BRAF600E targeting drugs, and BCL-2 inhibitors.

### Expression levels of IAPs, DRs and BCL-2 may guide for tailored targeted therapeutics

Analysis of expression levels of apoptotic factors in several colorectal (CRC) adenocarcinoma cell lines provides evidence for their potential importance as tumour markers and/or targets, complimenting previous reports. XIAP, DR4 and DR5 are overexpressed at the mRNA and protein levels in all adenocarcinoma cell lines. Notably, high levels of BCL-2 protein are detected in RKO adenocarcinoma cell line. This data were further exploited here towards developing rational and efficient preclinical protocols based on the oncogenic and apoptotic profile of the tumour cell lines.

### Birinapant and AT-406 show a mild effect on CRC tumour cells as mono-treatments

Treatment of a panel of CRC cell lines with novel SMAC-mimetics Birinapant and AT-406 resulted in a decrease of cell viability and cell migration properties, as well as appearance of apoptotic characteristics of selected colorectal adenocarcinoma cells, like the aggressive RKO cells. Regarding cell migration, IAPs can directly control Rho GTPases, thus regulating cell shape and migration (1). Here, for the first time, reduction of cancer cell migration by Birinapant is reported.

This study provides further evidence and interest on Birinapant, a novel SMAC-mimetic and promising anticancer agent [30] and its recently published proof of mechanism data for quantifying apoptotic biomarkers in clinical trials [31]. On the other hand, the mild treatment effects of the tested SMAC-mimetics propose for their rational combined treatment protocols in resistant cell lines, either with BRAFV600E inhibitors or with other therapeutics targeting apoptosis like TRAIL or BCL-2 inhibitors.

### Combined treatments of SMAC mimetics with BRAF inhibitor sensitise resistant BRAFV600E Colorectal tumour cells

BRAFV600E Colorectal tumours show intrinsic resistance to BRAFV600E specific inhibitors Vemurafenib and Dabrafenib (13), otherwise very efficient against BRAFmut melanoma (12). Many studies, including the current, aim at improving the efficiency of Vemurafenib against colorectal tumours by rational combined treatments.

Here, SMAC-mimetics Birinapant and AT-406 can synergise with PLX4720, a Vemurafenib lead compound, towards efficient antitumour treatments of BRAFV600E colorectal adenocarcinoma cells in 2D and 3D cultures. It is of interest, that the successful protocol includes pretreatment with Birinapant with subsequent Birinapant- PLX4720 combined treatment, which is necessary for tumour cell death to be induced.

These results support the therapeutic combination of Birinapant with multiple chemotherapies, as shown for those therapies that can induce TNF secretion [34]. Remarkably, recent studies have provided strong evidence that Birinapant co-treatment can overcome platinum resistance in a tumour-initiating subpopulation of ovarian cancer [35], as well as in a tumour-initiating AML subpopulation in combination with demethylating agents [36].

### TRAIL can synergise with SMAC mimetics to efficiently drive resistant tumour cells to apoptosis

Despite the fact that during colorectal carcinogenesis a marked increase in sensitivity to TRAIL has been reported, colorectal adenocarcinomas like HT29 and RKO remain partially resistant to TRAIL-induced apoptosis [37]. TRAIL resistance has been associated with defective ceramide signalling [38, 39]. Among the many studies of efficient combined treatments in colorectal cancer cells involving TRAIL: quercetin can enhance TRAIL-mediated apoptosis in colon cancer cells by inducing the accumulation of death receptors in lipid rafts [32] and the selective BRAF V600E inhibitor PLX4720 acts synergistically with TRAIL in order to overcome oncogenic PIK3CA resistance in colon cancer cells [33].

Here, treatment with SMAC-mimetics Birinapant and AT-406 can sensitize resistant-to-TRAIL and SMAC-mimetics HT29 cancer cells to apoptosis in 2D and 3D cultures. The combination of Birinapant and TRAIL leads to simultaneous activation of both the intrinsic and the extrinsic pathway. The same synergistic effect has been shown in the combined treatment of AT-406 and TRAIL. Inhibiting IAP function by SMAC-mimetics can apparently sensitise the HT29 TRAIL resistant cell line to TRAIL-Birinapant (or AT-406) combined treatments. These results are complementing those of other studies [40], where Birinapant in combination with TNF-a exhibits a strong anti-melanoma effect in vitro and enhances TRAIL potency in inflammatory breast cancer cells in an IAP-dependent and TNF-α-independent mechanism [41].

### Combined targeting of apoptosis at the BCL-2 and IAPs level is efficient for CRC cells

Early in this study, expression analysis of apoptotic factors in the panel colon adenocarcinoma cell lines has provided evidence for a notable overexpression of BCL-2 in RKO cell line. Therefore treatments with ABT-199, a specific inhibitor of BCL-2 were performed, which resulted in reduction of cell viability in RKO cell line.

ABT-199 is shown here to act synergistic with SMAC-mimetics Birinapant and AT-406 on cell viability of RKO colon adenocarcinoma cells. ABT-199 selectively targets BCL-2 not BCL-XL and is active as a single agent in lymphoid malignancies such as CLL and non-Hodgkin lymphoma [31].

Recently, a very efficient synergistic protocol of BRAF with autophagy inhibitors in colorectal cancer cells has been presented, as another example of the advantages and better efficiency of rational combined treatments as compared to mono-treatments [42].

## Conclusions

Efficient protocols of inhibition of IAPs activity and anti-apoptotic effect are presented by using Birinapant or AT-406 alone and in their combinations with either TRAIL (Figs. 5, 6 and 7) or with other inhibitors of pro-survival pathways, like BRAF-MEK (Fig. 4) and BCL-2 (Fig. 8). Synergistic rational anticancer combined protocols are presented depending on the tumour cell background, like resistance to individual treatments, BRAF mutation or BCL-2 overexpression. These rational combined treatments of agents targeting the tumour cell apoptotic machinery to restore some of its functions lost in cancer, can lead to efficient anti-cancer protocols, once further validated at the pre-clinical level and finally in the clinic.

## Abbreviations

BCL-2, B-cell lymphoma 2; BIRC, baculovirus IAP repeat; BRAF kinase, rapidly accelerated fibrosarcoma kinase; CRC, colorectal cancer; DISC, death-inducing signaling complex; IAPs, inhibitors of apoptosis proteins; PARP, poly (ADP-ribose) polymerase; RIP kinases, receptor-interacting protein kinases; SMAC, second mitochondria-derived activator of caspases; TRAIL, TNF-related apoptosis-inducing ligand

## Additional files

Additional file 1: Figure S3. Combined treatment of the other SMAC-mimetic AT-406 with PLX4720. (TIF 1.89 mb) Additional file 2: Figure S2a and Figure S2b. Treatments of Birinapant (0.5μΜ and 1μΜ) in combination with TRAIL on tumour cell viability. Figure S2c. Birinapant (10μΜ) synergistic effect in combination with TRAIL. (TIF 2.64 mb)
