# Supplementary figures and images for: IAP antagonists Birinapant and AT-406 efficiently synergise with either TRAIL, BRAF, or BCL-2 inhibitors to sensitise BRAFV600E colorectal tumour cells to apoptosis
Source: BMC Cancer. 2016 Aug 12;16:624. doi: 10.1186/s12885-016-2606-5 (PMC4982265; doi:10.1186/s12885-016-2606-5)

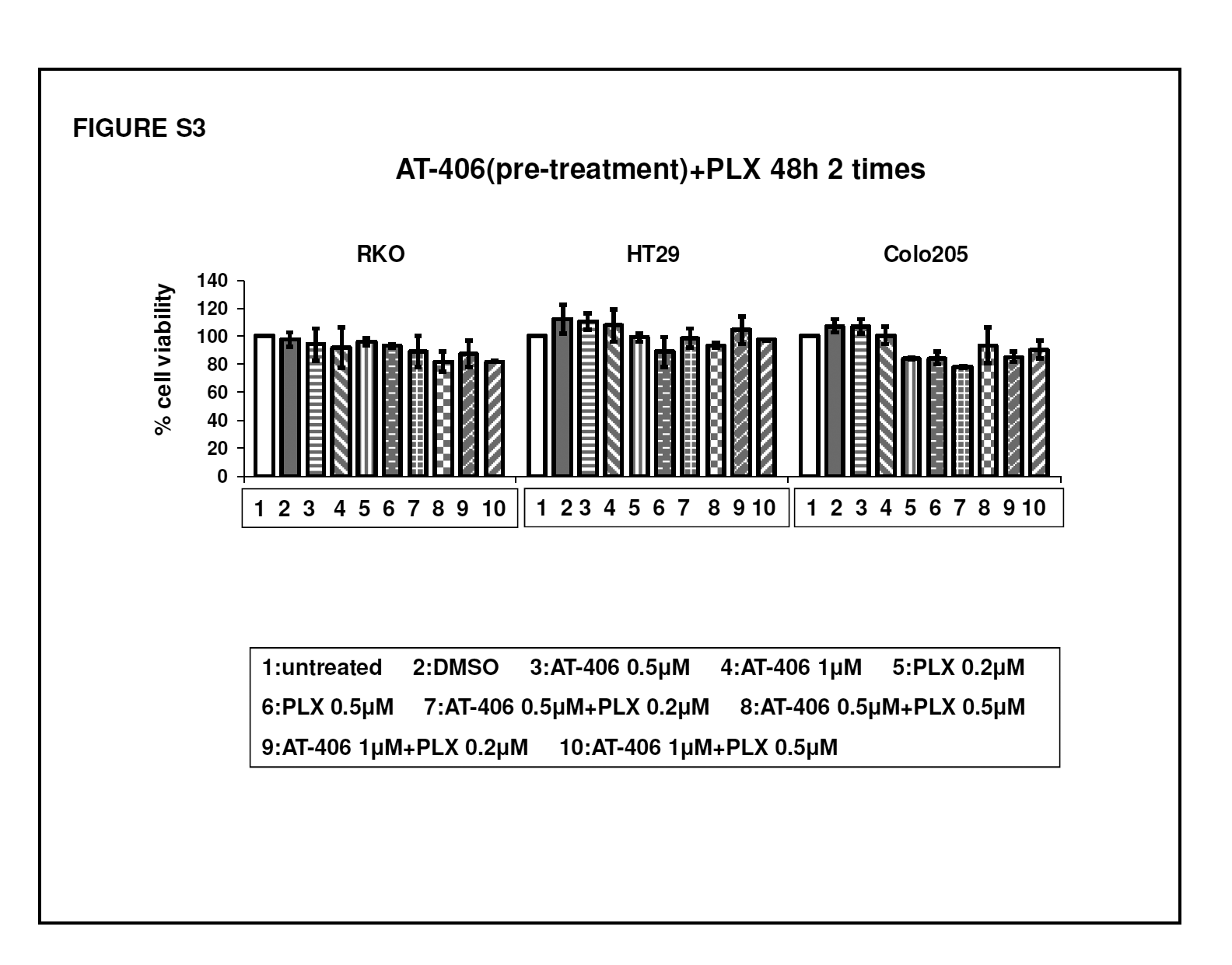

Supplement: Additional file 1: Figure S3. — Combined treatment of the other SMAC-mimetic AT-406 with PLX4720. (TIF 1.89 mb) [file 12885_2016_2606_MOESM1_ESM.tif]

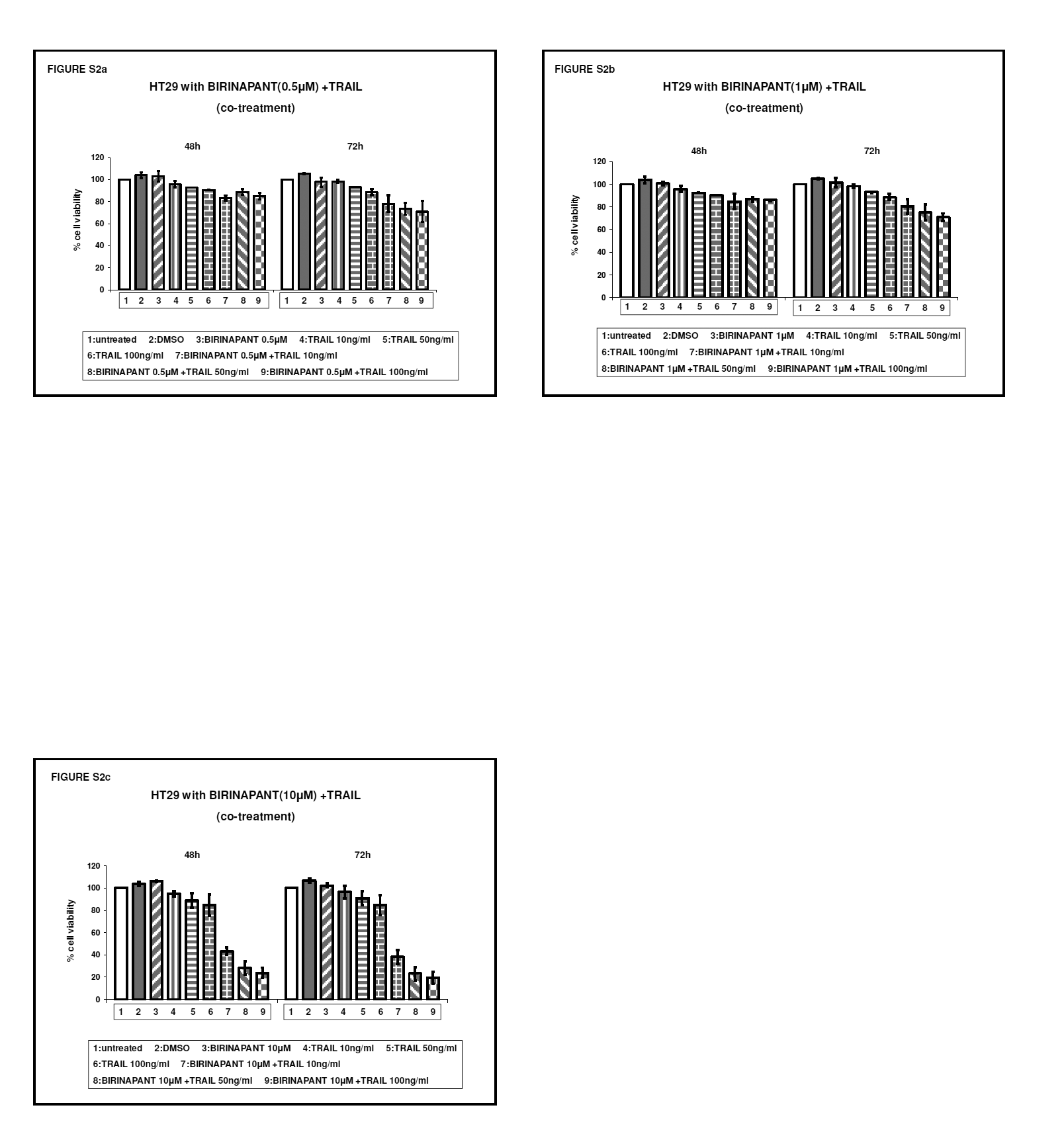

Supplement: Additional file 2: Figure S2a and Figure S2b. — Treatments of Birinapant (0.5μΜ and 1μΜ) in combination with TRAIL on tumour cell viability. Figure S2c. Birinapant (10μΜ) synergistic effect in combination with TRAIL. (TIF 2.64 mb) [file 12885_2016_2606_MOESM2_ESM.tif]
